# Histopathology of the *Plasmodiophora brassicae*-Chinese Cabbage Interaction in Hosts Carrying Different Sources of Resistance

**DOI:** 10.3389/fpls.2021.783550

**Published:** 2022-01-13

**Authors:** Xitong Liu, Stephen E. Strelkov, Rifei Sun, Sheau-Fang Hwang, Rudolph Fredua-Agyeman, Fei Li, Shifan Zhang, Guoliang Li, Shujiang Zhang, Hui Zhang

**Affiliations:** ^1^Institute of Vegetables and Flower, Chinese Academy of Agricultural Sciences, Beijing, China; ^2^Department of Agricultural, Food and Nutritional Science, University of Alberta, Edmonton, AB, Canada

**Keywords:** *Plasmodiophora brassicae*, Chinese cabbage, resistance, histopathology, primary infection, secondary infection

## Abstract

Clubroot is a serious soil-borne disease of crucifers caused by the obligate parasite *Plasmodiophora brassicae*. The genetic basis and histopathology of clubroot resistance in two Chinese cabbage (*Brassica rapa* ssp. *pekinensis*) inbred lines Bap055 and Bap246, challenged with pathotype 4 of *P. brassicae*, was evaluated. The Chinese cabbage cultivar “Juxin” served as a susceptible check. The resistance in Bap055 was found to be controlled by the *CRa* gene, while resistance in Bap246 fit a model of control by unknown recessive gene. Infection of the roots by *P. brassicae* was examined by inverted microscopy. Despite their resistance, primary and secondary infection were observed to occur in Bap055 and Bap246. Primary infection was detected at 2 days post-inoculation (DPI) in “Juxin,” at 4 DPI in Bap055, and at 6 DPI in Bap246. Infection occurred most quickly on “Juxin,” with 60% of the root hairs infected at 10 DPI, followed by Bap055 (31% of the root hairs infected at 12 DPI) and Bap246 (20% of the root hairs infected at 14 DPI). Secondary infection of “Juxin” was first observed at 8 DPI, while in Bap055 and Bap246, secondary infection was first observed at 10 DPI. At 14 DPI, the percentage of cortical infection in “Juxin,” Bap055 and Bap246 was 93.3, 20.0, and 11.1%, respectively. Although cortical infection was more widespread in Bap055 than in Bap246, secondary infection in both of these hosts was restricted relative to the susceptible check, and the vascular system remained intact. A large number of binucleate secondary plasmodia were observed in “Juxin” and the vascular system was disrupted at 16 DPI; in Bap055 and Bap246, only a few secondary plasmodia were visible, with no binucleate secondary plasmodia. The defense mechanisms and expression of resistance appears to differ between Chinese cabbage cultivars carrying different sources of resistance.

## Introduction

Clubroot, caused by the obligate parasite *Plasmodiophora brassicae* Wor., is a major soil-borne disease of the Brassicaceae. Clubroot represents a major threat to cruciferous vegetable production in Canada, China, India, Europe, and Australia (Bhattacharya et al., 2014; Chai et al., 2014; Donald and Porter, 2014; Rahman et al., 2014; Wallenhammar et al., 2014). In China, the disease was first reported in Taiwan and Fujian in the 1910s, and now occurs widely spread across the country (Chai et al., 2014). Transmission of *P. brassicae* occurs on seeds, in soil, infected plant material, irrigation water and animal manure, with the most severe clubroot outbreaks reported in the southwest, northeast and middle regions of China. The disease is estimated to cause yield losses of 20–30% in Chinese cabbage annually (Wang et al., 2011; Chai et al., 2014).

Isolates of *P. brassicae* are classified into pathotypes based on their virulence patterns on various host differential sets. Pathotype 4, as defined on the system of Williams (1966), is prevalent in most of China (Chai et al., 2014). The clubroot pathogen produces long-lived resting spores, which can remain viable in the soil for many years and hinder management of the disease. Given the importance of clubroot as a disease of Chinese cabbage and other brassicas, *P. brassicae* has become an urgent problem for breeders, growers, and farmers (Dixon, 2009). Various strategies are recommended for clubroot management, including the sanitization of field implements and equipment, the application of soil amendments and chemical pesticides, and long rotations out of susceptible hosts (Hwang et al., 2014; Peng et al., 2014a; Andreote et al., 2020). The most economical and effective approach for clubroot control, however, is to breed varieties with genetic resistance to the disease (Diederichsen et al., 2009; Rahman et al., 2011; Peng et al., 2014b). The identification, mapping and cloning of resistance genes serve as the basis for rapid selection of new clubroot-resistant (CR) varieties. At present, more than 20 resistance gene loci have been mapped, which are found mainly on chromosomes A01, A02, A03, A06, and A08. Among the mapped resistance genes, *CRa*, *CRb^kato^*, and *Crr1* have been cloned (Ueno et al., 2012; Hatakeyama et al., 2013, 2017), and all are R genes with an NBS-LRR structure (Eitas and Dang, 2010).

The resting spores of *P. brassicae* germinate to produce primary zoospores, which initiate infection by invading the root hairs (Tommerup and Ingram, 1971; Kageyama and Asano, 2009; Liu et al., 2020b). The zoospores encyst on the root hairs, piercing the cell wall and injecting their contents into the cytoplasm of the host cell (Aist and Williams, 1972). Inside the root hairs, *P. brassicae* forms primary plasmodia. A number of nuclear divisions occur synchronously in these plasmodia, followed by cleavage into zoosporangia. Later, 4–16 secondary zoospores are formed in each zoosporangium and released back into the soil. The secondary zoospores penetrate the cortical tissues of the main roots, a process called secondary infection. At this stage, the pathogen colonizes the underground parts of plants, reprogramming existing meristematic activities to form nutrient sinks as well as creating favorable conditions for resting spore formation (Malinowski et al., 2012). Secondary infection is responsible for the characteristic symptoms of clubroot on susceptible hosts, including the hypertrophy and hyperplasia associated with root gall formation (Kageyama and Asano, 2009). When root galling is severe, aboveground plant growth is severely affected. Inside the root, secondary plasmodia develop into a new generation of resting spores, which are eventually released back into soil as survival structures as the galls decompose (Tommerup and Ingram, 1971; Ingram and Tommerup, 1972; Kageyama and Asano, 2009).

*Plasmodiophora brassicae* infects vegetables and other hosts in the Brassicaceae family, and serves as a useful model system with which to study the disease (Koch et al., 1991; Mithen and Magrath, 1992; Ludwig-Müller et al., 2009). It is thought that perturbations in phytohormone content, particularly auxin and cytokinin (Siemens et al., 2011), in *P. brassicae*-infected plants play important roles in disease development, but little is known regarding the changes that occur in different resistant hosts that do not develop typical root galling. A better understanding of these processes may provide strategies to improve plant tolerance to infection.

Recently, Liu et al. (2020a) reported that cortical infection was restricted in the clubroot-resistant European Clubroot Differential ECD 10 (*Brassica napus var. napobrassica*) and ECD 04 (*B. rapa* ssp. *rapifera*), but the mechanisms by which this occurred were not examined. While there has been progress in understanding the molecular interactions between *P. brassicae* and its hosts (Chen et al., 2019; Ciaghi et al., 2019; Liu et al., 2020a), microscopy-based comparisons of the infection and resistance processes in hosts carrying different sources of resistance have been lacking. In this study, the genetic basis of clubroot resistance was compared in two CR recombinant inbred lines of Chinese cabbage, Bap055 and Bap246, using gene linkage markers and genetic analysis, and the histopathology of the resistance response was evaluated by inverted microscopy.

## Article Types

Disease Resistance for Sustainable Agriculture.

## Materials and Methods

### Plant and Pathogen Material

Two clubroot-resistant (CR) Chinese cabbage inbred lines Bap055 and Bap246 (*B. rapa*, 2n = 2x = 20), (Chinese cabbage, heading, spring type), developed by the Institute of Vegetables and Flowers, Chinese Academy of Agricultural Sciences, were included in this study, along with the clubroot-susceptible Chinese cabbage cultivar “Juxin,” which served as a control.

To evaluate the genetic control of resistance, Bap055 and Bap246 were used as resistant male parents to cross with a susceptible female parent Bac1344, a *B. rapa* inbred line. Two crosses Bac1344 × Bap055, Bac1344 × Bap246 were made under greenhouse conditions. Subsequently, two F_1_ individual plants from each cross were used to make a backcross to the susceptible parent (Bac1344) to produce BC_1_ populations with selfing to produce F_2_ populations. Further phenotypic evaluation was performed in each of these BC_1_ and F_2_ populations to determine the genetic control of resistance of Bap055 and Bap246.

The field isolate of *P. brassicae* used in this study was collected from infected Chinese cabbage plants growing in a farm in Beijing, China, in 2019, and was classified as pathotype 4 on the differential system of Williams (1966; Supplementary Table 1). The isolate was stored as resting spores in galled root tissue at −20°C until needed.

### Inoculation

To prepare resting spore suspensions of *P. brassicae*, 50 g of galled root tissue was removed from storage and allowed to thaw at room temperature before being homogenized in a blender in 600 mL of distilled water. The homogenate was filtered through eight layers of cheesecloth, and the resting spore concentration in the filtrate was estimated using a hemocytometer and adjusted to 1 × 10^8^ spores/mL with sterile distilled water. The spore suspensions were prepared immediately prior to use.

Seedlings were inoculated by the root dip method of Johnston (1968) with some modifications. Briefly, the seeds of the three host genotypes were placed on a single layer of moistened filter paper in Petri dishes, and allowed to germinate at room temperature for 3–4 days before inoculation. The rootlets were then soaked in a Petri dish containing the *P. brassicae* resting spore suspension for about 10 min, non-inoculated host filled with distilled water, and transferred into 50 cell trays (54 cm × 28 cm × 6 cm), at a rate of one seedling per cell, filled with a sterilized potting mix (2 parts peat soil: 1 part perlite: 1 part vermiculite). All plants were kept under controlled conditions at 21–23°C and 16 h light and 8 h darkness in a greenhouse, with watering and fertilization as required.

### Disease Evaluation and Statistical Analysis

**FIGURE 1:**
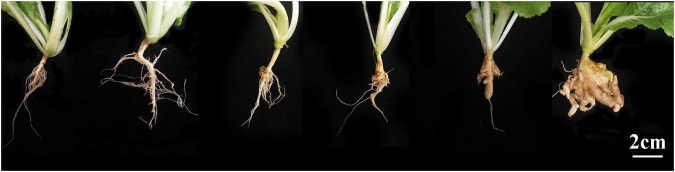
Illustration of the clubroot severity rating scale. From left to right are 0, 1, 3, 5, 7, and 9.

Six weeks after inoculation, the seedlings were removed from the trays and the roots were cleaned with water for assessment of clubroot severity on 0, 1, 3, 5, 7, and 9 disease severity scale based on technical specifications for the identification of clubroot resistance in cruciferous vegetables (DB36/T765-2013; Zhang et al., 2019), where: 0 = no clubs; 1 = a few small clubs on the lateral or main root; 3 = clubs on the main root where the diameter was <2× the base of the stem, and there were a few small clubs on the lateral roots; 5 = club(s) on the main root with a diameter 2–3× the base of the stem and moderate clubbing on the lateral roots; 7 = club(s) on the main root with a diameter of 3–4× the base of the stem, and severe clubbing on the lateral roots; and 9 = the root system almost without lateral roots, club(s) on the main root with a diameter >4× the base of the stem, and crack(s) observed on the club (Figure 1). Scores of 0 and 1 were regarded as indicative of resistance, while scores of 3, 5, 7, and 9 were considered susceptible responses.

The individual severity ratings were used to calculate a disease index (DI) using Eq. 1 (Yang et al., 2020):


$$\mathrm{DI}=\frac{\left(0\times n\,0\right)+\left(1\times n\,1\right)+\left(3\times n\,2\right)+\left(5\times n\,3\right)+\left(7\times n\,4\right)+\left(9\times n\,5\right)}{\left(N\times 9\right)\times 100}$$

Where *n*0 indicates the number of plants rated as 0, *n*1 indicates the number of plants rated as 1, *n*2 indicates the number of plants rated as 3, *n*3 indicates the number of plants rated as 5; *n*4 indicates the number of plants rated as 7, *n*5 indicates the number of plants rated as 9, and N is the total number of plants evaluated. In addition, a disease incidence was calculated as per Eq. 2:


$$\mathrm{Disease}\,\mathrm{Incidence}=\frac{n\,2+n\,3+n\,4+n\,5}{N}\times 100\%$$

The disease index (DI) of each host was calculated based on the mean value of all tested seedlings. DI = 0, immune; 0 < DI ≤ 10, highly resistant (HR); 10 < DI ≤ 30, resistant (R); 30 < DI ≤ 50, susceptible (S); DI > 50, highly susceptible (HS).

### Analysis of Markers Linked to CR Genes and Polymerase Chain Reaction

The hosts were screened for the presence of CR loci/genes (*CRa, CRc, CRk, CRd, Crr1, Crr2, Rcr1*, and *CrrA5*) using 29 linked markers (Suwabe et al., 2003, 2006; Hirai et al., 2004; Piao et al., 2004; Saito et al., 2006; Hayashida et al., 2008; Sakamoto et al., 2008; Matsumoto et al., 2012; Ueno et al., 2012; Chen et al., 2013, 2016; Hatakeyama et al., 2013; Kato et al., 2013; Chu et al., 2014; Zhang et al., 2014; Pang et al., 2018). Total genomic DNA was isolated from the leaves using a cetyltrimethylammonium bromide (CTAB) method (Saghai-Maroof et al., 1984) and subjected to polymerase chain reaction (PCR) analysis in a Veriti Thermal Cycler (Thermo Fisher Scientific). Reactions were carried out in a 10 μL containing 25 ng of each primer 30 ng of genomic DNA (Supplementary Table 2), and 5 μL of 2 × PCR Master Mix. The PCR conditions consisted of an initial denaturation step at 95°C for 5 min, followed by 35 cycles of 95°C for 15 s, 55°C for 15 s, and 72°C for 1 min and a final extension for 7 min at 72°C. Amplification products were resolved on a standard agarose (2%) or polyacrylamide (6%) gel and their sizes were compared with those expected for the specific resistance genes. Selected amplicons were extracted from the gels using linked marker Craim-T and sent to Sangon Biotech for sequencing to confirm their identities.

### Preparation and Observation by Hand-Sectioning

To compare the progress of primary and secondary infection, inoculated roots of the two clubroot resistant lines and the susceptible cultivar “Juxin” were examined every 2 days using an inverted microscope (ZEISS Axio, Germany). Images were captured with a Canon camera (EOS R6, Japan) on the microscope. Sections were prepared following Ellison et al. (2016) with minor modifications. Briefly, the roots were washed with sterile water and placed into a 15 mL centrifuge tube containing 10 mL of FAA fixative (formalin: acetic acid: 70% ethyl alcohol = 5: 5: 90). After 48 h at 4°C, the FAA fixative was removed and the samples were washed with 70% ethanol for 5 min. They were the cut into 1-cm sections, and sealed with water for rapid observation of the sections. Five individual plants were examined at each time-point for each host genotypes, with three roots observed from each plant. For observation of the primary infection stage, 100 root hairs were observed per plant, repeated three times. The percentage of infected root hairs was calculated as the number of infected root hairs/100 root hairs × 100%. For the observation of cortical (secondary) infection, three microscopic fields of view were observed for each plant (five plants from each material), repeated three times and the number of cortical infections was recorded. The percentage of infected cortices was calculated as the number of infected cortices/45 cortices × 100%.

### Preparation and Observation of Paraffin Sections

To observe the effects on the root structure Bap055, Bap246 and “Juxin” following infection, paraffin sections were prepared. The roots were washed gently with sterile water. Then the main root was fixed in FAA (formalin: acetic acid: 70% ethyl alcohol = 5: 5: 90) and kept in the fixative for at least 2 days. Paraffin sections were prepared according to Ruzin (1999). The roots were dehydrated in an ethanol series (75, 85, 90, and 95% ethanol for 4 h, 2 h, 2 h, and 1 h, respectively), followed by anhydrous ethanol twice for 30 min each time. The roots were then transferred to a 1/2 absolute ethanol + 1/2 xylene mixture and 100% xylene for 10 min each, respectively, followed by paraffin embedding and sectioning. Sections (5 mm thick) were cut with a microtome and stained with Safranin O and Fast Green following Zhang et al. (2017). Briefly, the wax was removed by washing the sections twice in 100% xylene for 15 min each time. The sections were then placed in a solution of 1/2 absolute ethanol + 1/2 xylene for 5 min, followed by a graded ethanol series of 100% ethanol (5 min), 100% ethanol (5 min), 95% ethanol (2 min), and 85% ethanol (2 min). The sections were then stained for at least 12 h in 1% w/v Safranin O (75% ethanol), washed in 85% ethanol for 5 min, and then counter-stained with Fast Green 0.05% w/v (95% ethanol) for 10–15 s. The sections were placed in a 1/2 absolute ethanol + 1/2 xylene mixture for 5 min and cleared by washing twice in 100% xylene (5–10 min per wash). The sections were mounted with Permount (Fisher Chemical) and observed and photographed in a Nikon Eclipse 50i microscope equipped with a Nikon DS-Fi1 digital camera. The other paraffin sections were stained with Aniline Blue/Toluidine Blue (fixation and methods were the same as above), dyed with Aniline Blue/Toluidine Blue for about 30 min, then washed with tap water, dehydrated with ethanol, cleared with xylene and sealed with neutral gum. They were then observed and photographed with a Nikon Eclipse 50i microscope with a Nikon DS-Fi1 digital camera.

### Statistical Analysis

Replicated observations were made randomly and independently of each other and had a normal distribution with common variances. Thus, the assumption of ANOVA was generally met. A one-way ANOVA was performed to determine the amounts of infected root hairs and cortical tissue using SPSS v. 16.0 (SPSS Inc., Chicago, IL, United States). The least significant difference (LSD) method was used to test significance, and differences were considered to be significant at *P* < 0.05 unless otherwise noted.

## Results

### Clubroot Incidence and Severity

**FIGURE 2:**
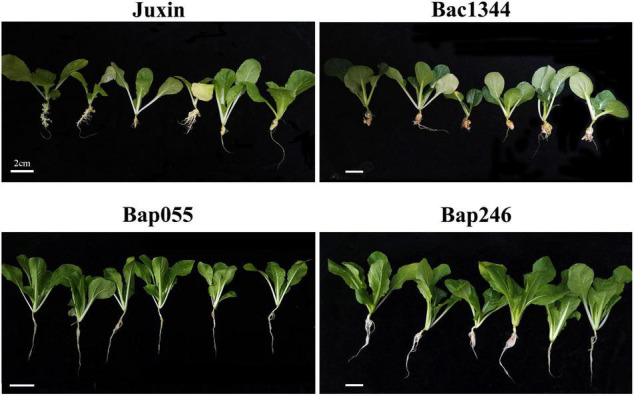
Clubroot resistance assay on four genotypes. Three-day-old seedlings of each material were inoculated with resting spores of *P. brassicae*, and all plant roots were collected at 6 weeks and assayed for the clubroot severity.

The Chinese cabbage genotypes Bap055, Bap246, Bac1344, and “Juxin” were inoculated with *P. brassicae* the field isolate from Beijing, China (pathotype 4), and assessed for clubroot development after 6 weeks. The roots of “Bac1344” and “Juxin” had developed severe galling (DIs = 98 and 91, respectively; Table 1), with some roots beginning to decompose, resulting in plant death (Figure 2). Disease incidence on Bac1344 and “Juxin” was 99.3 and 97.7%, respectively, and was not significant difference between the genotypes (Table 1). In contrast, few or no galls were observed on the roots of Bap055 and Bap246 (Figure 2), with these hosts developing low levels of visible clubroot (DIs = 8.8 and 5.3, respectively; Table 1).

**TABLE 1:** Disease index of Chinese cabbage hosts in greenhouse trials and the percentage of infected cortex.

Host	Disease incidence (%)	Disease index (DI)	Resistance grade	The percentage of infected cortex (%)		
				10 days	12 days	14 days
Bap055	2.1	8.82	HR	8.9	17.8	20.0
Bap246	1.5	5.29	HR	5.6	8.6	11.1
Bac1344	99.3	98.12	HS	–	–	–
Juxin	97.7	91.25	HS	20	55.6	93.3

*HR, highly resistant; HS, highly susceptible.*

### Genetic Analysis of Different Clubroot-Resistant Hosts

To investigate the genetic basis of resistance to clubroot in Bap055 and Bap246, the susceptible pak choi inbred line Bac1344 was crossed with Bap055 and Bap246 to obtain the F_1_ generations separately. The F_1_ was backcrossed with the susceptible parent Bac1344 to obtain the BC_1_ population, and the F_1_ selfed to obtain the F_2_ populations. The populations were inoculated and evaluated for clubroot development.

All thirty-two F_1_ plants from the cross of Bap055 × Bac1344 were resistant to clubroot. The ratio of resistance to susceptibility in the F_2_ population was 262:80, and exhibited a 3:1 segregation ratio at *P* < 0.05 (χ^2^ = 0.47 < 3.84). Of the 35 BC_1_ individuals, 16 were resistant and 19 were susceptible, exhibiting a 1:1 segregation ratio at *P* < 0.05 (χ^2^ = 0.26 < 3.84) (Table 2). The Indel maker Craim-T (Ueno et al., 2012) linked to *CRa* exhibited polymorphism in Bap055 and Bac1344, respectively. Collectively, these results indicated that a single dominant gene *CRa* controlled the resistance in Bap055.

**TABLE 2:** Genetic analysis of clubroot resistance in Bap055 and Bap246.

Material	Sum	Level						Disease index (DI)	Tested ratio	χ^2^	χ^2^_0.05_
		0	1	3	5	7	9				
Bap055	30	27	0	0	0	3	0	7.78	–	–	3.84
Bac1344	29	0	0	0	1	7	21	93.10	–	–	
F_1_	32	30	2	0	0	0	0	0.007	–	–	
BC_1_	35	11	5	5	3	8	3	–	1R:1S	0.26	
F_2_	342	176	86	15	13	30	22	–	3R:1S	0.47	
Bap246	48	39	9	0	0	0	0	3.03	–		
Bac1344	33	0	0	0	3	7	23	91.25	–		
F_1_	35	0	0	0	5	5	25	90.48	–		
BC_1_	36	0	0	0	2	2	32	–	–		
F_2_	849	130	76	85	136	203	219	–	1R:3S	0.25	

All thirty-five F_1_ plants from the cross of Bap246 × Bac1344 were susceptible. Among 849 F_2_ plants, the ratio of resistance to susceptibility in the F_2_ population was 206:643, and exhibiting a 1:3 segregation ratio at *P* < 0.05 (χ^2^ = 0.25 < 3.84). The 36 BC_1_ plants were all susceptible (Table 2). These results indicated that the clubroot resistance in Bap246 was controlled by unknown recessive gene.

### Microscopic Observation of Primary Infection in Different Hosts

**FIGURE 3:**
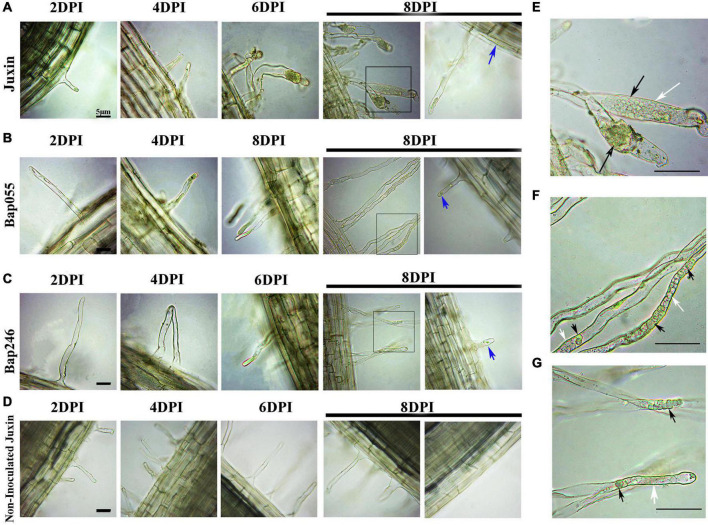
Differences in the primary infection were investigated in the host genotypes Bap055, Bap246, and “Juxin” at 2–8 days post-inoculation (DPI). **(A)** Inverted microscopy images showing primary infection of “Juxin” at 2–8 DPI. **(B)** Primary infection of Bap055 at 2–8 DPI. **(C)** Primary infection of Bap246 at 2–8 DPI. **(D)** Inverted microscopy images of non-infected “Juxin” at 2–8 days, no invasion of *P. brassicae.* The part highlighted with a black box in the lower panel was further enlarged for a view in **(E–G)**. **(E)** Multinucleate zoosporangial plasmodia at “Juxin” was profiled. **(F)** Multinucleate zoosporangial plasmodia at Bap055 was profiled. **(G)** Multinucleate zoosporangial plasmodia at Bap246 was profiled. Zoosporangia with content are indicated with black arrows, while some empty zoosporangia without any content are indicated with white arrows, blue arrows indicate secondary zoospores.

The progress of infection by *P. brassicae* in the dominant resistant host Bap055, the recessive resistant host Bap246, and the susceptible host “Juxin” was compared by microscopy. Non-inoculated “Juxin” were included as a control. Sections were made to observe infected root hairs under an inverted microscope, which indicated the presence of several lipid droplet-enriched uninucleate primary plasmodia in Bap055 and Bap246 as well as in “Juxin” (Figures 3A–C). Primary infection was detectable in “Juxin” at 2 DPI, but was not observed in Bap055 and Bap246 until 4 and 6 DPI, respectively (Figures 3A–D). At 6 DPI, zoosporangia were observed in the root hairs of “Juxin,” where the root hair tips were enlarged, and each zoosporangium in the root hairs had multiple nuclei (Figure 3A). The same structures were observed in Bap055 and Bap246 at 8 DPI (Figures 3B,C), along with some empty zoosporangia indicating release of the secondary zoospores (Figures 3E–G). Free uninucleate secondary zoospores were also observed (Figures 3B,C). These finding suggest that *P. brassicae* can initiate and complete primary infection of Bap055 and Bap246, but that infection is delayed in both Bap055 and Bap246 relative to “Juxin,” and is slowest in Bap246.

### The Amount of Early Infection and Secondary Infection in Different Hosts

**FIGURE 4:**
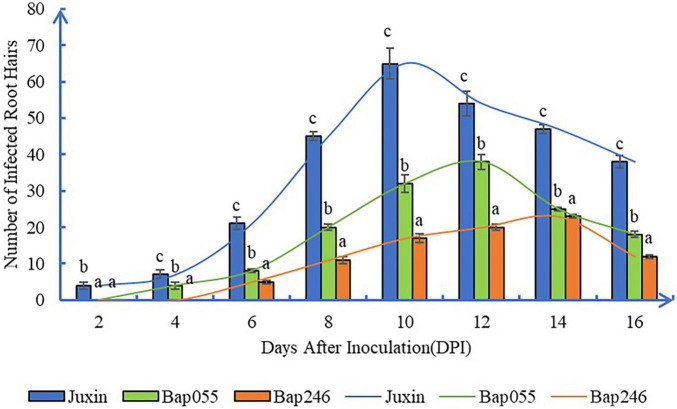
Root hair infection rate in three Chinese cabbage genotypes at different time-point after inoculation of *P. brassicae*. The curves showed similar infection rate trends in the three hosts, at first increased, and then decreased overtime. Data are mean ± SE. Different letters on the same color bars indicate significant difference at *P* < 0.05 level by LSD test.

Primary infection rates showed similar trends in the clubroot-susceptible “Juxin” and the resistant lines Bap055 and Bap246 (Figure 4). Infection rates at first increased, and then decreased overtime. However, the infection rates in Bap055 and Bap246 were significantly lower than in “Juxin” at 6–16 DPI, with infection lowest in Bap246. Peak infection rates were observed at 10 DPI in “Juxin,” 12 DPI in Bap055 and 14 DPI in Bap246, when root hair infection reached 60, 31, and 20% in each of the hosts, respectively.

**FIGURE 5:**
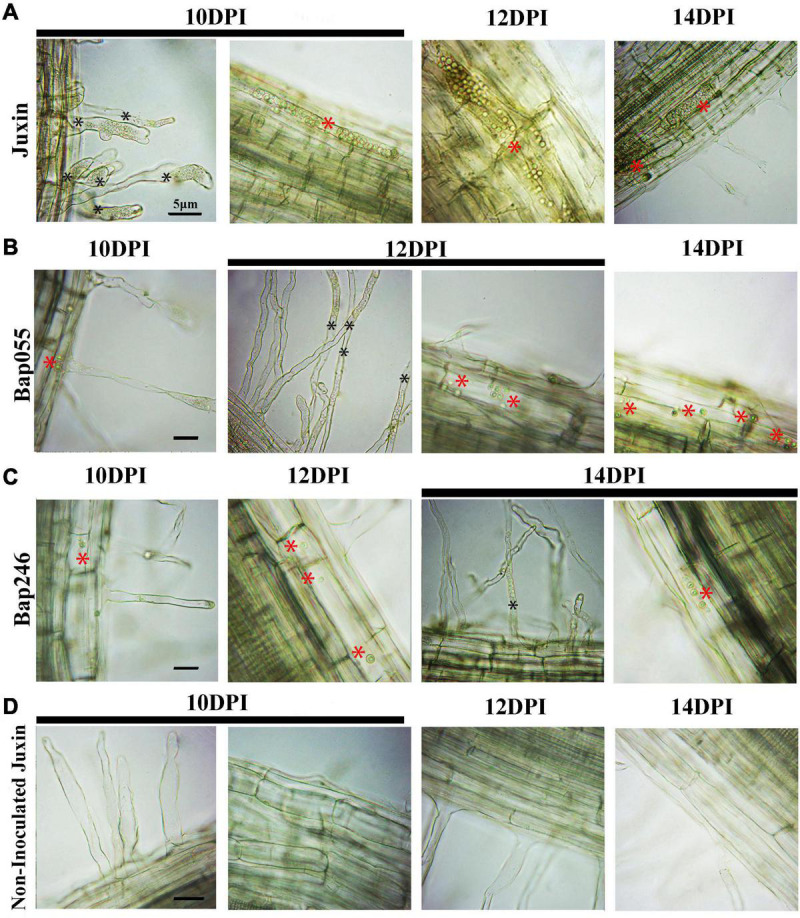
Secondary infection of different Chinese cabbage genotypes by *P. brassicae* at 10–14 days post-inoculation (DPI). **(A)** Inverted microscopy images of cortical cells of the genotype “Juxin” at 10–14 DPI. **(B)** Inverted microscopy images of cortical cells of the genotypes Bap055 at 10–14 DPI. **(C)** Inverted microscopy images of cortical cells of the genotype Bap246 at 10–14 DPI. **(D)** Inverted microscopy images of cortical cells of non-infected “Juxin”. Secondary plasmodium of host cortical cells was indicated with red *, Zoosporangia of root hair were indicated with black *.

At 8 DPI, secondary zoospores had infected the cortex of “Juxin,” indicating the beginning of secondary infection (Figure 3A). In contrast, secondary zoospores were not observed in the cortex of Bap055 and Bap246 until 10 DPI (Figure 5). Secondary infection rates were also different among the host genotypes, particularly at 10–14 DPI. While root cortical infection increased over the time-course across all three hosts, secondary infection rates were 93.3% in “Juxin,” 20.0% in Bap055, and 11.1% in Bap246 (Table 1). Based on the analysis of freehand root sections, infected root hairs and uninucleate secondary zoospores were much more abundant in the cortex of the susceptible host “Juxin” than in the CR hosts Bap055 and Bap246, in which few secondary zoospores were observed (Figure 5).

### Microscopic Observation of Secondary Infection of Different Hosts

**FIGURE 6:**
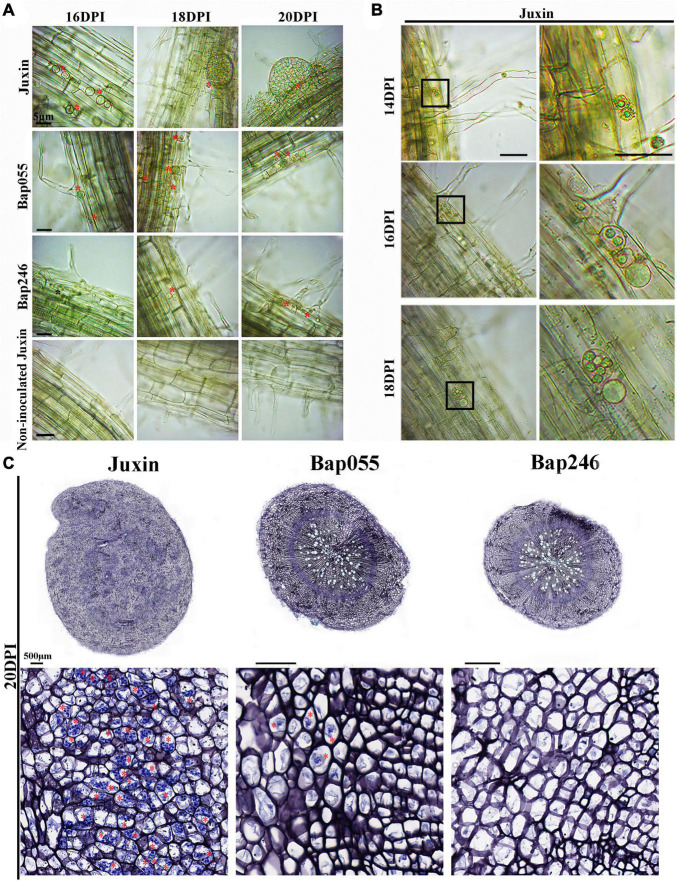
Secondary infection of different Chinese cabbage genotypes by *P. brassicae* at 16–20 days post-inoculation (DPI). **(A)** Inverted microscopy images of cortical cells of genotypes “Juxin”, Bap055, Bap246, and non-inoculated “Juxin” at 16–20 DPI, showing multinucleate secondary plasmodia. **(B)** The changes in secondary plasmodium in “Juxin” at 14–18 DPI, uninucleate secondary plasmodia at 14 DPI, binucleate secondary plasmodia at 16 DPI, and multinucleate secondary plasmodia at 18 DPI. The areas highlighted within the black boxes in the lower panel are enlarged for more detail. **(C)** Root sections of “Juxin,” Bap055, and Bap246 at 20 DPI. The areas highlighted within black boxes are enlarged for more detail. Multinucleate secondary plasmodia are indicated with red asterisks (*), while uninucleate secondary plasmodia are indicated with black asterisks.

While secondary zoospores invaded the cortex of Bap055 and Bap246, the growth and development of *P. brassicae* in the two CR hosts was restricted to varying degrees after the start of the secondary infection. At 16–20 DPI, many multinucleate secondary plasmodia appeared in the cortex of “Juxin,” while the development of *P. brassicae* in the cortex of Bap055 and Bap246 was extremely slow or did not progress (Figure 6). At 16 DPI, the pathogen had formed binucleate and multinucleate secondary plasmodia in “Juxin” (Figures 6A,B), while in Bap055 multinucleate secondary plasmodia were just beginning to form. The development of secondary plasmodia in Bap246 lagged further behind, and was still at the uninucleate secondary plasmodial stage at 16 DPI (Figure 6A). At 18 DPI, galls had begun to develop on the roots of “Juxin,” with a proliferation and expansion of secondary plasmodia in the cortex to form very obvious multinucleate secondary plasmodia (Figures 6A,B). In contrast, no galls were visible on Bap055 and Bap246 at 18 DPI, although a few round multinucleate secondary plasmodia were observed in the cortex (Figure 6A). At 20 DPI, the roots of “Juxin” continued to swell, and the secondary plasmodia in the cortex further multiplied and divided further until the cells were filled with *P. brassicae* (Figures 6A,C). In the two clubroot-resistant hosts, very few, small and round multinucleate secondary plasmodia (and no binuclear secondary plasmodia) were observed (Figures 6A,C).

### Microscopic Observation of Root Structure Associated With Secondary Infection in Different Hosts

**FIGURE 7:**
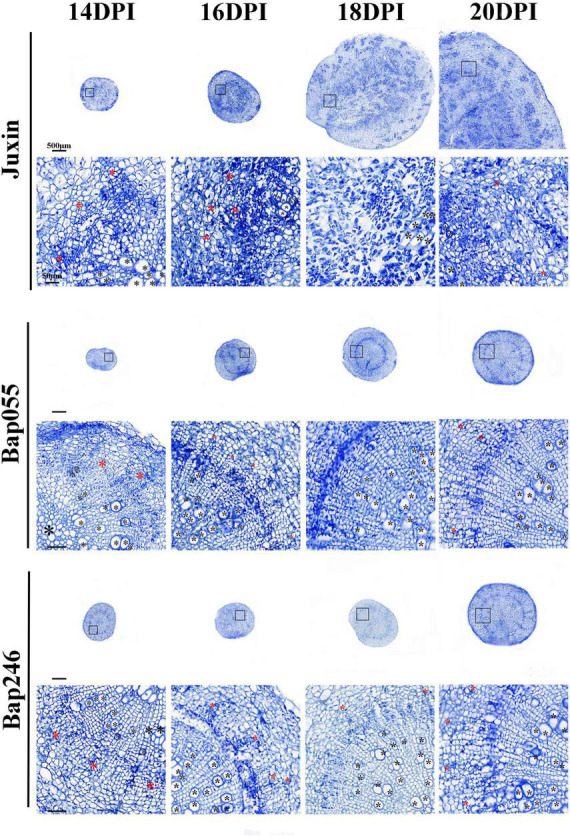
Paraffin root sections of the Chinese cabbage genotypes “Juxin,” Bap055, and Bap246 at 14–20 days post-inoculation (DPI) with *P. brassicae*. The areas highlighted within the black boxes are enlarged for more detail. Xylem is indicated with black asterisks (*) and phloem is indicated with red asterisks.

**FIGURE 8:**
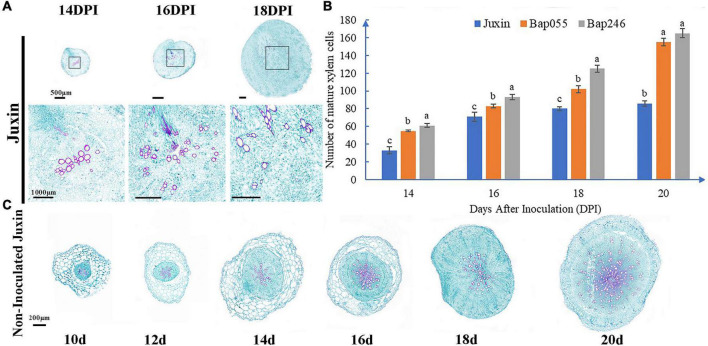
Changes of root of “Juxin” in secondary infection stage at 14–20 days post-inoculation (DPI). **(A)** Root sections of the clubroot susceptible Chinese cabbage host “Juxin” at 14–18 DPI with *P. brassicae*. The roots of “Juxin” were disrupted from 16 DPI. The areas highlighted within the black boxes are enlarged for more detail. **(B)** Number of mature xylem cells in “Juxin,” Bap055, and Bap246 at 14–20 DPI. The formation of new xylem was inhibited from the onset of gall formation from 16 DPI onward of “Juxin.” **(C)** Root sections of non-inoculated “Juxin” at 10–20 DPI. The root grows normally without structural change. Different letters on the same color bars indicate significant difference at *P* < 0.05 level by LSD test.

The roots of “Juxin” were disrupted from 16 DPI due to morphological changes and cell division intensified associated with infection, while the roots of Bap055 and Bap246 did not exhibit abnormal changes (Figures 7, 8). Paraffin sections stained with Safranin Fast Green were made to observe when and how the root structure of “Juxin” changed at 14–18 DPI, where in the xylem stained red and the phloem and other cells stained green (Figure 8A). While no abnormal phloem or xylem cells were visible at 14 DPI, the root structure had changed at 16 DPI. The positions of xylem cells were scattered, and the roots appeared “hollow” and abnormally enlarged. Xylem cells were more numerous, but were constricted and smaller due to the excessive division of the phloem (Figure 8A). As infection progressed, cell division became more widespread across the hypocotyl and swollen host cells that contained plasmodia were evident. The formation of new xylem was inhibited from the onset of gall formation from 16 DPI onward were observed (Figure 8B), and at later stages of gall formation (20 DPI onward) only small fragments of xylem were observed, with the vascular cambium (VC) became fragmented and characteristic islands of cell division present (Figures 7, 8A). Phloem formation in “Juxin” was not inhibited during the proliferative stages of gall development, although it was disordered. No abnormal changes in the root structure of non-infected “Juxin” at this stage (Figure 8C).

## Discussion

At present, most of the clubroot resistance genes that have been identified are mainly derived from the A genome of European turnip (Mehraj et al., 2020). This resistance is complete and controlled by major gene(s), including *Crr1a* and *CRa* (Ueno et al., 2012; Hatakeyama et al., 2013). Despite a seemingly ample collection of resistance loci, the identification of novel sources of resistance is often necessary. *P. brassicae* shows physiologic specialization, so the host’s resistance genes often confer immunity to only subsets of pathotypes, and a single resistance gene may be rapidly overcome. The loss of effectiveness of clubroot resistance has been reported from many regions worldwide (Diederichsen et al., 2014; Strelkov et al., 2016). Therefore, in this study, we compared two hosts showing high levels of resistance, but under differential genetic control. The resistance in Bap055 was controlled by *CRa* as confirmed with molecular markers, while the resistance in Bap246 fit a model of control by unknown recessive gene. This allowed for a follow-up analysis of the relationship between *P. brassicae* infection and different resistant hosts. Further exploration of the clubroot resistance mechanisms in different resistant hosts may be valuate for the breeding of plant materials with wide resistance. *P. brassicae* is distinct from other plant pathogens, such as fungi or oomycetes, as it is a typical obligate soilborne protist parasite of the Brassicaceae (Schwelm et al., 2015; Pérez-López et al., 2020). Compared with ectoparasitic pathogens, different clubroot-resistant materials may possess different cellular and molecular mechanisms of resistance to *P. brassicae.* Only basic and preliminary explorations have been conducted in this line of research.

### Stages of *P. brassicae* Blocked in the Resistant Hosts During Its Life Cycle

Liu et al. (2020b) refined the life cycle of *P. brassicae* in susceptible host plants of Arabidopsis. In this study, we found that *P. brassicae* follows a similar infection process in the susceptible host “Juxin.” Therefore, the life cycle of *P. brassicae* proposed in Arabidopsis appears to represent a general model for most Brassicaceae. In this study, we identified the stages of the *P. brassicae* life cycle that are blocked in two resistant *B. rapa* hosts. The pathogen could initiate primary infection in the two resistant hosts, produce zoosporangial plasmodia in the root hair and epidermis, and continue to grow and develop to produce and release a large number of secondary zoospores, hence completing the primary infection stage. Secondary zoospores could penetrate the cell wall and enter the cortical cells. However, further growth and development of the secondary zoospores appeared limited to a certain extent in the cortical cells of the resistant hosts, leading to an interruption of the secondary infection consistent with the results of Deora et al. (2012) and Yuan et al. (2021). The formation of multinucleate secondary plasmodia was rarely observed in the resistant hosts in the later period, and no resting spores were noted.

### Primary Infection and Secondary Infection Were Not Completely Separated in Time

The infection stages of *P. brassicae* are not completely separated in time and space. Primary infection and secondary infection can occur simultaneously (Jiang et al., 2020). The present study indicated that secondary infection occurred in resistant and susceptible hosts, while primary infection continued, with the number of infected root hairs increasing slowly or even plateauing or decreasing. As secondary infection progressed, differences in pathogen development were observed, with high proportions of empty zoosporangia, uninucleate and multinucleate secondary plasmodia, low proportions multinucleate primary plasmodia, zoosporangia, and uninucleate primary plasmodia. The development *P. brassicae* appeared highly asynchronous, consistent with the results of Liu et al. (2020b).

### *Plasmodiophora brassicae* Completes the Primary Infection Phase in Different Clubroot-Resistant Hosts

The role of primary infection by *P. brassicae* is to insert a single primary zoospore into the host root hair or epidermis to form a zoosporangium and release a large number of secondary zoospores, facilitating the establishment of secondary infection (Feng et al., 2013a,b). The present study indicated that the clubroot pathogen could complete the entire primary infection stage in two CR hosts, producing a number of secondary zoospores. It appears that to a certain extent, the CR hosts could not recognize the invading *P. brassicae* and prevent its growth and development in root hairs and epidermal cells. This is different from the resistance of the plant host to extracellular pathogens, which can show effective prevention of pathogen invasion directly on the plant epidermis (Schwelm et al., 2015).

Plants can recognize potential pathogens *via* two perception systems (Chisholm et al., 2006; Jones and Dangl, 2006). The first detects conserved microbial molecules, termed pathogen- or microbe-associated molecular patterns (PAMPs or MAMPs), through pattern recognition receptors (PRRs), leading to PAMP-triggered immunity (PTI). The other evolved to recognize specific microbial virulence effectors, usually through intracellular resistance proteins (R proteins), resulting in effector-triggered immunity (ETI). Jones and Dangl (2006) proposed a zigzag model, which posits that in host-pathogen interactions, plants can use their own evolved receptor proteins to identify pathogens *via* PAMPs and trigger PTI. In order to infect plants successfully, pathogens must then secrete effector molecule(s) to inhibit PTI and the plant defense response. Eventually, plants initiate a new round of defense gene expression, which can recognize the effector molecules secreted by pathogens and trigger ETI, thereby further preventing the infection and expansion of pathogens. It is likely that *P. brassicae* can overcome PTI and invade the root hairs of the resistant *B. rapa* hosts, while ETI is not completely triggered at this time, enabling progress of the primary infection stage. In the future, it would be enlightening to study how the clubroot pathogen can avoid or inhibit the plant monitoring systems in other hosts with different types of genetic resistance.

### Primary Infection Delayed to Varying Degrees in Clubroot-Resistant Hosts

The speed of primary infection by *P. brassicae* can vary. In this study, primary infection of the susceptible host was observed at 2 DPI, consistent with the results of Macfarlane (1958) and Hwang et al. (2011). However, primary infection in the *B. rapa* lines carrying dominant and recessive resistance was not detected until 4 and 6 DPI, respectively, and the extent for root hair infection was significantly reduced relative to the susceptible host. Hence, primary infection progressed more quickly and was more widespread in the susceptible vs. resistant hosts.

Studies have shown that R genes found in natural plant populations were used early, and that domestication favored dominant R genes providing full resistance (Kou and Wang, 2010). Nonetheless, recessive R genes and R genes that provide partial resistance may provide more durable resistance to plant pathogens (Kourelis and van der Hoorn, 2018). Clubroot-resistant hosts with dominant/recessive R genes may have different resistance mechanisms and express at different times, resulting in different primary infection levels.

Cao et al. (2018) found that resistance to *Xanthomonas oryzae* pv. *oryzae* (*Xoo*) conferred by dominant disease resistance (MR) genes and recessive MR genes resulted in different types of programmed cell death (PCD). In the current study, it is possible that the recessive resistant host deployed a stronger clubroot-resistance mechanism earlier, so that primary infection by *P. brassicae* was delayed more strongly. In the future, transcriptome analysis can be performed on dominant resistant, recessive resistant and susceptible hosts during early infection, to explore the mechanisms of different resistance types and analyze the changes in expression levels of different transcription factors at different times.

Although the extent of root hair infection was greatest in the susceptible host, root hair infection rates showed similar trends of increasing first and then decreasing across the three hosts, with the infection peak rate observed at 10–14 DPI. After *P. brassicae* invades the root hairs, the plasmodium cleaves to form secondary zoospores, which are discharged into the soil environment. We found that the decline of root hair infection in the susceptible host occurred earlier than in the resistant hosts, so the former may enter the secondary infection stage earlier. While secondary zoospores can infect root hairs again (McDonald et al., 2014), the root hair infection rate did not rise again in this experiment. This may reflect release of the secondary zoospores from the root hairs more quickly than primary and secondary zoospores could invade.

### Secondary Infection by *P. brassicae* in the Clubroot-Resistant Hosts Was Blocked

The results of this study indicated that *P. brassicae* completed the primary infection stage in the two resistant *B. rapa* hosts, and that the secondary zoospores produced following primary infection invaded the cortex. Similarly, Zhang et al. (2015) reported that secondary infection occurred in clubroot susceptible and resistant hybrid canola cultivars by single-spore isolate. This may reflect the main pathotype from the field isolate invade the cortex of clubroot-resistant hosts.

Nonetheless, while secondary infection was observed in the CR hosts in the present study, *P. brassicae* growth and development in the root cortex appeared to be inhibited. While many CR gene loci have been reported in various brassicas, only *Crr1a* and *CRa* have been cloned and functionally verified, having the NBS-LRR structure. These R genes that belong to a large multi-gene family that can be separated into two subclasses, the toll-interleukin-1 (TIR) class and the coiled-coil (CC) class (Rafiqi et al., 2009). The dominant resistant host in this study carried the *CRa*, which belong to the TIR-NBS-LRR protein domain family (Ueno et al., 2012). In plants, this domain triggers defense responses following perception of pathogen effectors (Chisholm et al., 2006; Jones and Dangl, 2006; Rafiqi et al., 2009; Dodds and Rathjen, 2010). These responses include localized cell death, necrosis, destruction of cell wall or secondary thickening of the xylem as part of the hypersensitive response (HR), which may be associated with host resistance against *P. brassicae* (Dekhuijzen, 1979; Fuchs and Sacristán, 1996; Donald et al., 2006; Jiang et al., 2020). Nonetheless, evidence for a classical HR in the clubroot pathosystem is limited, and no localized cell death or other changes associated with this reaction were observed in the CR hosts in the current study.

The basis of clubroot resistance in the recessive resistant host is unknown. The secondary plasmodia in the cortex of this host were smaller and less numerous. Auxin (IAA) and cytokinin (CK) regulate the growth, development and division of *P. brassicae* (Davies, 2010; Ludwig-Müller, 2014), while the salicylic acid (SA) defense signal pathway plays an important role in this plant-pathogen interaction (Djavaheri et al., 2019). The recessive resistant host may strongly inhibit the activation of IAA and CK pathways earlier, and rapidly activate various SA and other defensive pathways to upregulate several pathogenicity-related proteins (PRPs) to block development of *P. brassicae*. More work is needed to understand the different resistance mechanisms to the clubroot pathogen.

### Secondary Infection Had No Effect on the Vascular System of Resistant Hosts

While secondary infection by *P. brassicae* was detected in the CR *B. rapa* hosts in this study, there was no change in the root structure, with the vascular system developing normally. In contrast, biotrophic life history of *P. brassicae* renders significant changes to elucidating morphological and cellular characteristics of *P. brassicae* development in host tissues during clubroot disease initiation and development (Tu et al., 2019). The vascular system of the susceptible host was severely distorted. The xylem was constricted by cells and became smaller and scattered. Phloem cells continued to divide abnormally and the walls of some cells were broken at 16 DPI. Within the clubroot galls there was an increased formation of phloem cells and an arrest of xylogenesis. Earlier studies have found that the secondary plasmodia in susceptible hosts proliferate and expand in the root tissue, inducing irregular growth of the root tissue, leading to a disintegration of the centrosymmetric root structure and the destruction of the vascular system (Kobelt et al., 2000). Similarly, gall formation was reported to disrupt vascular development, with a significant reduction in xylem, increase meristematic activities within the vascular cambium (VC) and phloem parenchyma (PP) cells in the region of the hypocotyl (Malinowski et al., 2012; Walerowski et al., 2018). Secondary infection is the main cause of visible clubroot symptoms (Tommerup and Ingram, 1971; Ingram and Tommerup, 1972; Kageyama and Asano, 2009; Liu et al., 2020a), so the prevention and treatment of clubroot should include measures that are effective as soon as possible prior to 16 DPI.

The vascular system of plants is a complex tissue system composed of phloem, intermediate cambium, and xylem. Phloem and xylem play a special role, transporting water, nutrients, metabolites and small signaling molecules, allowing vascular plants to adapt to changing environments (Lacombe and Achard, 2016). At the same time, the tissue is rich in nutrients and can provide survival conditions for various pathogens. Many vascular-related pathogens are tissue specific and rely on this tissue to obtain essential nutrients (Fukuda and Ohashi-Ito, 2019; Xian et al., 2020). Changes in vascular system differentiation has implications for host–pathogen interactions in clubroot, as *P. brassicae* is an obligate biotrophic parasite that establishes a strong sink for carbohydrates to supply nutrients during gall formation (Keen and Williams, 1969; Mitchell and Rice, 1979; Evans and Scholes, 1995).

Lignin not only contributes to the immune response of plants, but also provides an important physical barrier to limit pathogen infection and is one of the important components of vascular resistance (Chezem and Clay, 2016; Karasov et al., 2017; Mehraj et al., 2020). When resistant hosts are infected by *P. brassicae*, the activity of lignin-related synthase may increase, leading to an increase in lignin content, strengthening of the cell walls, and further stabilization of the vascular system for protection from *P. brassicae*. Given the difficulties in culturing *P. brassicae in vitro* and challenges in studying its direct effects on the vascular bundles, current knowledge on the host defense mechanisms involved is limited (Ohtani et al., 2017; Jiang et al., 2019).

## Conclusion

This study has provided some information on the genetic basis of resistance to *P. brassicae* in CR Chinese cabbage lines, indicating that this resistance was controlled by *CRa* in one host, and by as a yet unidentified recessive gene in another. Histological examinations confirmed that while primary infection progressed in both CR hosts, it was slower and less widespread than in a susceptible check cultivar. Similarly, while secondary infection did occur, it did not progress to the formation of a new generation of resting spores in the CR lines. These results indicate the importance of identifying and comparing different sources of resistance, as well as of complementing genetic and molecular studies with microscopy-based evaluations to track the host-pathogen interaction, thereby improving our understanding of the control and basis of genetic resistance to clubroot.

## Data Availability Statement

The original contributions presented in the study are included in the article/Supplementary Material, further inquiries can be directed to the corresponding author.

## Author Contributions

HZ and SJZ were responsible for the conceptualization and design of the experiment. XL was responsible for performing the experiments and conducting the work. HZ and XL were involved in writing this manuscript. FL and RS participated in the preparation and editing of the manuscript. SS, S-FH, and RF-A were responsible for analyzing the data and revising the manuscript. HZ, SFZ, and GL were responsible for providing advice on the studies and revising this work. All authors contributed to the article and approved the submitted version.

## Conflict of Interest

The authors declare that the research was conducted in the absence of any commercial or financial relationships that could be construed as a potential conflict of interest.

## Publisher’s Note

All claims expressed in this article are solely those of the authors and do not necessarily represent those of their affiliated organizations, or those of the publisher, the editors and the reviewers. Any product that may be evaluated in this article, or claim that may be made by its manufacturer, is not guaranteed or endorsed by the publisher.

## Supplementary Material

Supplementary Figure 1 Difference in CRa between Bap246 and Bac1344. CRa appeared as an amplification polymorphism. The sequences of primer used are given in Supplementary Table 2.
