# Supplementary figures and images for: Histopathology of the Plasmodiophora brassicae-Chinese Cabbage Interaction in Hosts Carrying Different Sources of Resistance
Source: Front Plant Sci. 2022 Jan 13;12:783550. doi: 10.3389/fpls.2021.783550 (PMC8792839; doi:10.3389/fpls.2021.783550)

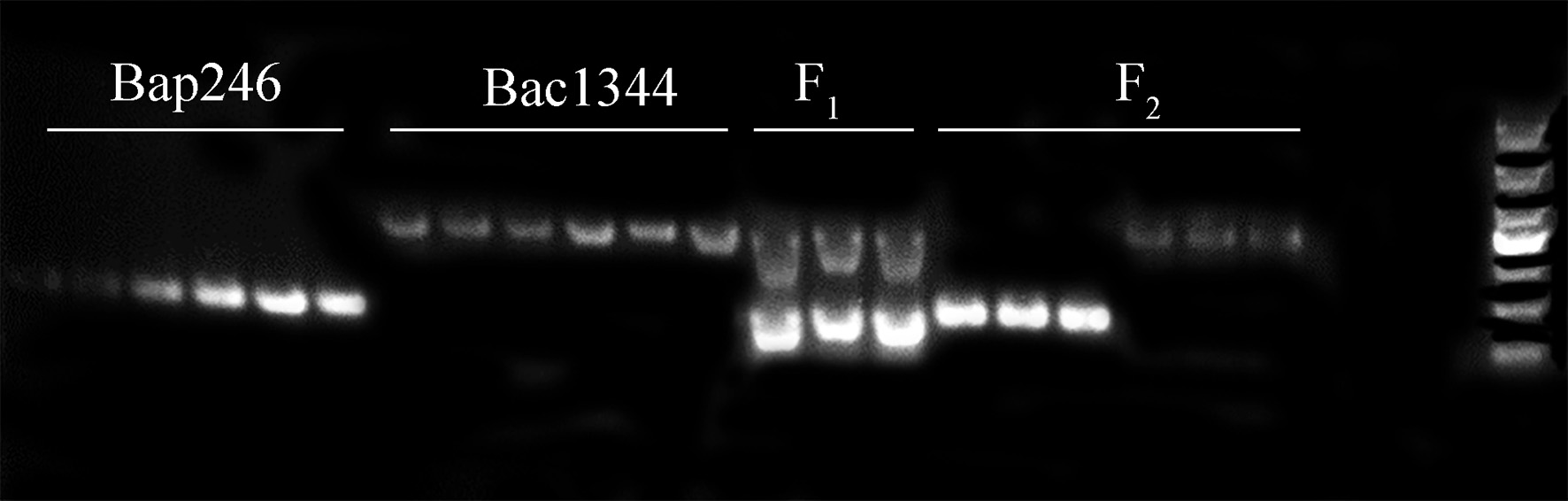

Supplement: Supplementary Figure 1 — Difference in CRa between Bap246 and Bac1344. CRa appeared as an amplification polymorphism. The sequences of primer used are given in Supplementary Table 2. [file Image_1.JPEG]
