# Supplementary material for: Histopathology of the Plasmodiophora brassicae-Chinese Cabbage Interaction in Hosts Carrying Different Sources of Resistance
Source: Front Plant Sci. 2022 Jan 13;12:783550. doi: 10.3389/fpls.2021.783550 (PMC8792839; doi:10.3389/fpls.2021.783550)
Supplement: Supplementary file 2 [file Table_1.DOCX]

**Supplemental Table 1** Identification of pathotypes of *P. brassicae* on the differential hosts of Williams (1966)

| Field Isolate  Origin | Differential Host and Disease Index* | | | | | Pathotype |
| --- | --- | --- | --- | --- | --- | --- |
|  | Number | Jersey Queen | Badger Shipper | Laurentian | Wilhelmsburger |  |
| Shunyi, Beijing, China | Ⅰ | 63.2 | 89.3 | 73.2 | 79.2 | 4 |
|  | Ⅱ | 78.8 | 72.8 | 90.1 | 85.9 | 4 |
|  | Average | 80.0 | 81.1 | 81.7 | 82.6 | 4 |

*The pathotype test was conducted twice (I and II); the index of disease obtained for each run of the experiment for each host is indicated.
