# Supplementary material for: Histopathology of the Plasmodiophora brassicae-Chinese Cabbage Interaction in Hosts Carrying Different Sources of Resistance
Source: Front Plant Sci. 2022 Jan 13;12:783550. doi: 10.3389/fpls.2021.783550 (PMC8792839; doi:10.3389/fpls.2021.783550)
Supplement: Supplementary file 3 [file Table_2.DOCX]

**Supplymentary Table 2** Linked markers used in screening of known clubroot-resistant genes

| **Primer IDs** | **Gene** | **Sequence of primer F1(5^,^-3^,^)** | **Sequence of primer R1(5^,^-3^,^)** | **Reference** |
| --- | --- | --- | --- | --- |
| Craim-T | CRa | TATATTAATGATAAAGCAGAAGAAGAAA | AATGCGACTGAGAAAGTTGTAG | Ueno *et al.*, 2012 |
| GC1680 |  | ATTCTACTTCCTTTTAGCTGCTCTAACCGC | TTCGTAATAATTGATTTGCATATCAATTGG | Ueno *et al.*, 2012 |
| GC2360-1 |  | CAGCACCAGCATAACCAGCTACAGTC | AGAACTTTGCAAGTGGCTCAGATAAT | Ueno *et al.*, 2012 |
| SC2930-T |  | TAGACCTTTTTTTTGTCTTTTTTTTTAC | CTAAGGCCATAGAAATCAGGTC | Matsumoto et al.,2012 |
| SC2930-Q |  | CAGACTAGACTTTTTGTCATTTAGACT | AAGGCCATAGAAATCAGGTC | Matsumoto et al.,2012 |
| HC352b-SCAR |  | CTTTATAATGGCTACTATTTA | TGCTCATGAGTGTATAACTA | Hayashida et al.,2008 |
| TCR79 | CRb | TGACGTTCAATCAAAGCCTGA | TTTAGCAATCAAATGCAAATTCAA | Zhang et al.,2014 |
| TCR108 |  | CGGATATTCGATCTGTGTTCA | AAAATGTATGTGTTTATGTGTTTCTGG | Zhang et al.,2014 |
| K-3 |  | CTTTGGATTGTTGACCTT | ATGTTGATGCTACTGAGAC | Chen et al，2016 |
| TCR09 |  | GCAGCAACCGATAATATAAGGA | AACCAGAAGAAGAAAAACAAAAA | Piao *et al.*, 2004 |
| TCR05 |  | AGAATCATGACCGGGGAAAT | GCAGCTAAGTCATCGACCAA | Piao *et al.*, 2004 |
| KBrH129J18R | CRb^Kato^ | AGAGCAGAGTGAAACCAGAACT | GTTTCAGTTCAGTCAGGTTTTTGCAG | Kato et al.,2013 |
| B50 | CRc | GATTCAATGCATTTCTCTCGAT | CGTATTATATCTCTTTCTCCATCCC | _ |
| m6r |  | CCTCTTGGAAAACCCATGAA | GCAATTATTGGCCTGTTCGT | Sakamoto *et al.*, 2008 |
| B50-C9 |  | GATTCAATGCATTTCTCTCGAT | CGTATTATATCTCTTTCTCCATCCC | Matsumoto et al.,2012 |
| B50-6R |  | AATGCATTTTCGCTCAACC | CGTATTATATCTCTTTCTCCATCCC | Matsumoto et al.,2012 |
| HC688-4-6 | CRk | TCTCTGTATTGCGTTGACTG | ATATGTTGAAGCCTATGTCT | Matsumoto et al.,2012 |
| HC688-4-7 |  | TCTCTGTATTGCGTTGACTG | AAATATATGTGAAGTCTTATGATC | Matsumoto et al.,2012 |
| yau376 | CRd | TGTCACCAGCGCATTATAGC | AAGGGAGGGAAGATGGGTTG | Pang et al. 2018 |
| yau389 |  | TCAAGTGGAGAGGGTCAGTC | CAGTCACGAGGTCCCTAACA | Pang et al. 2018 |
| In185 | Crr1 | TCTTTCTGGATGCTCTAGCC | TATATCCGACCTGTACCAAGCAG | _ |
| BRMS-088 |  | TATCGGTACTGATTCGCTCTTCAAC | ATCGGTTGTTATTTGAGAGCAGATT | Suwabe *et al.*, 2003 |
| BRMS-096 | Crr2 | AGTCGAGATCTCGTTCGTGTCTCCC | TGAAGAAGGATTGAAGCTGTTGTTG | Suwabe *et al.*, 2003 |
| OPC11-2S | Crr3 | GTAACTTGGTACAGAACAGCATAG | ACTTGTCTAATGAATGATGATGG | Hirai *et al.*, 2004 |
| OPC11-1S |  | TTACAGCTGGACCAAGAACATAG | ATCGATGTTTGTGAGTCTCTACT | Hirai *et al.*, 2004 |
| BrSTS-54 |  | CGTATAGACATAGAAGACATGGAAGC | GTGTTTATGCTGATTCCTTCACAG | Saito *et al.*, 2006 |
| BrSTS-61 |  | CCATCCGCAGGAGTTAAAGTTGTA | AGAAAACGGCTGAGGGTTACTAC | Saito *et al.*, 2006 |
| sN8591 | Rcr1 | TTGTGGGCAGGAACAATACA | CTGGACGAGCCAAGCTAATC | Chu M et al., 2014 |
| Sr6340I |  | ATAGTTGGGAATGTGGCTGC | CGGACACGAAATCAAACCTT | Chu M et al., 2014 |
| BrCr-026 | CrrA5 | TCATCGATCCAATCCGTAA | CACGCAGTTAGAGTATCAAAGG | _ |
